# Post-Treatment High-Risk HPV Testing as a “Test of Cure”: A Systematic Review

**DOI:** 10.3390/medsci14050561

**Published:** 2026-09-11

**Authors:** Gavrila Timis, Romeo Micu, Adriana Oana Oltean, Iulian Goidescu, Renata Nicula, Lucian Constantin Mocan

**Affiliations:** 1 Doctoral School, “Iuliu Hatieganu” University of Medicine and Pharmacy, Victor Babes Square 8, 400347 Cluj Napoca, Romania; timis.gavrila@elearn.umfcluj.ro; 2 Department 2, Faculty of Nursing and Health Sciences, “Iuliu Hatieganu” University of Medicine and Pharmacy, 400012 Cluj Napoca, Romania; romeo.micu@umfcluj.ro (R.M.); oltean_adriana_ioana@yahoo.com (A.O.O.); 3 Obstetrics and Gynaecology I, Mother and Child Department, “Iuliu Hatieganu” University of Medicine and Pharmacy, 400006 Cluj Napoca, Romania; goidescu.iulian@elearn.umfcluj.ro; 4 Department of Surgery, “Iuliu Hatieganu” University of Medicine and Pharmacy, Croitorilor Square 19, 400006 Cluj Napoca, Romania; mocan.lucian@umfcluj.ro

**Keywords:** human papillomavirus, cervical intraepithelial neoplasia, test of cure, conization, LEEP, residual disease, recurrence, post-treatment surveillance, resection margins, HPV genotyping

## Abstract

**Background/Objectives:** Excisional treatment is effective for cervical intraepithelial neoplasia grade 2 or 3 (CIN2–3), but residual or recurrent high-grade disease remains clinically important. This focused systematic review evaluated post-treatment high-risk human papillomavirus (hrHPV) testing as a test of cure and compared it with cytology and resection-margin status. **Methods:** PubMed/MEDLINE was searched and supplemented by structured citation checking and targeted verification, with supplementary Embase and CENTRAL searches conducted during peer review; the review was not prospectively registered. Mixed-grade cohorts were retained only when a high-grade subgroup was extractable or for supportive qualitative evidence. **Results:** Fifteen primary studies enrolled 21,932 treated women, of whom 18,992 contributed to endpoint-specific analyses. Explicit CIN2+/CIN3+/HSIL+ event rates were 1.9–6.8% in most studies, with 11.8% in one earlier intensively verified cohort; higher rates in other reports reflected any-grade CIN or composite outcomes and were not treated as CIN2+. In the subset reporting directly comparable accuracy measures, hrHPV testing was generally more sensitive than cytology and negative predictive values were 98.0–100%. These values require caution because recurrence prevalence was low and histological verification was incomplete in several cohorts. Five studies were judged at low, eight at unclear, and two at high overall risk of bias for their diagnostic-accuracy contributions. **Conclusions:** The evidence supports hrHPV-based risk stratification and reduced-intensity surveillance after a negative test, but not unqualified return to routine population screening. Standardised prospective studies with uniform CIN2+ endpoints and complete verification remain needed.

## 1. Introduction

Cervical cancer remains one of the most common malignancies affecting women worldwide and a leading cause of cancer death among women, with the greatest burden falling on low- and middle-income countries [1,2,3]. Virtually all cases are attributable to persistent infection with high-risk human papillomavirus (hrHPV) genotypes, a causal relationship that has been documented beyond reasonable doubt and that underpins all contemporary prevention strategies [4,5]. The natural history of the disease proceeds through a well-characterised sequence of precancerous lesions—cervical intraepithelial neoplasia (CIN) grades 1 to 3, increasingly described under the two-tier squamous intraepithelial lesion terminology—before progression to invasive carcinoma. Moderate and severe dysplasia, corresponding to CIN2 and CIN3 and grouped clinically as high-grade squamous intraepithelial lesion (HSIL), represent the principal targets of secondary prevention because they carry an appreciable risk of progression if untreated [6]. The clinical question addressed in this review concerns not the detection of these lesions but what happens after they are surgically removed, and how best to confirm that treatment has succeeded.

The natural history of high-grade CIN is heterogeneous, and the probability of progression versus regression depends strongly on the causative genotype, with HPV-16 conferring the highest risk of progression to cancer [6]. This heterogeneity is the reason that, although a proportion of CIN2 lesions regress spontaneously, treatment of histologically confirmed high-grade disease is generally recommended once the diagnosis is established, particularly for CIN3 [6]. Excisional treatment—most commonly the loop electrosurgical excision procedure (LEEP), also termed large loop excision of the transformation zone (LLETZ), and cold-knife conization—has become the standard therapeutic approach for high-grade CIN because it removes the transformation zone while preserving a histological specimen for margin assessment [7]. Reported cure rates after a single excisional procedure are high, typically exceeding 90%, and the procedure can usually be performed in an ambulatory setting with minimal morbidity. The histological specimen also allows assessment of the resection margins, which has long been recognised as an indicator of the completeness of excision and of subsequent risk [7].

Nevertheless, treatment is not invariably curative. Reported post-treatment event rates depend strongly on the endpoint: high-grade CIN2+/CIN3+/HSIL+ is less frequent than composite outcomes that also include CIN1 or persistent hrHPV infection. Incomplete excision, reflected by involvement of the resection margins, increases the risk of post-treatment high-grade disease several-fold [7]. Treated women remain at elevated risk of invasive cervical cancer for many years, which justifies structured, risk-based follow-up rather than simple discharge to routine population screening. The prolonged excess risk, together with the imperfect performance of any single follow-up test, has motivated research into how best to identify treatment failure while avoiding unnecessary repeat procedures.

For much of the modern screening era, post-treatment surveillance relied on repeated cytology, sometimes supplemented by colposcopy, performed at intervals over one to two years. However, cytology has well-recognised limitations of sensitivity, particularly in the post-treatment cervix where scarring, distorted anatomy, and sampling difficulty can compromise interpretation [8]. The recognition that persistent hrHPV infection is the necessary cause of cervical neoplasia transformed the conceptual basis of follow-up: if the causal infection has been cleared, the biological driver of recurrence is removed, whereas persistence of hrHPV signals continuing risk [4,5]. Comparative diagnostic evidence has consistently shown that hrHPV testing is more sensitive than cytology for detecting high-grade disease, albeit at some cost to specificity, both in primary screening and in the triage and follow-up settings [8,9]. This insight gave rise to the concept of the hrHPV “test of cure” (ToC)—the use of a post-treatment hrHPV test, alone or combined with cytology, to stratify women into those who can safely return to routine screening and those who require intensified surveillance or further investigation.

Several specific controversies motivate a focused synthesis of this literature. First, there is the question of whether hrHPV testing alone is sufficient as a test of cure or whether co-testing with cytology adds clinically meaningful information; co-testing can increase test positivity and downstream colposcopy referrals [8]. Second, resection-margin status may help stratify follow-up intensity, although post-treatment hrHPV testing generally predicts treatment failure more accurately than margins [7]. Third, type-specific persistence may improve risk stratification: Heymans et al. found that persistence of the same high-risk genotype increased specificity and positive predictive value, although sensitivity decreased compared with pooled hrHPV testing [10]. Genotyping should therefore be considered promising rather than established as uniformly superior. Fourth, additional histological factors such as endocervical crypt involvement have been examined as potential predictors even when margins are clear, with conflicting results [11]. Fifth, the optimal timing of the first post-treatment test—6 months, 12 months, or intraoperatively—remains debated.

The clinical stakes of resolving these questions are considerable. Repeated or deep excisional procedures are associated with adverse obstetric outcomes, so unnecessary re-treatment of women who are cured carries real costs [12]. Against this backdrop, and in an era of HPV-based screening and vaccination [5,13], accurate confirmation of cure is important. Kocken et al. compared hrHPV testing, cytology, and co-testing for post-treatment CIN2+ [14], and Arbyn et al. subsequently synthesised 97 studies including 44,446 women [15]. The 2025 meta-analysis by Bomans et al. further updated pooled test performance across 46 studies and 20,385 women [16]. The present review does not seek to supersede those meta-analyses; it provides an individual-study, outcome-grade-stratified update that incorporates recent registry and biomarker cohorts and makes explicit which studies contribute to CIN2+-specific estimates versus supportive any-grade or virological evidence.

## 2. Materials and Methods

### 2.1. Study Design and Setting

This focused systematic review was reported in accordance with PRISMA 2020 [17]. The population comprised women treated by excision for histologically confirmed CIN2–3/HSIL; the index test was post-treatment hrHPV testing; comparators included cytology and resection-margin status; and the primary outcome was histologically or registry-confirmed residual/recurrent CIN2+. Mixed-grade cohorts were eligible only when a high-grade subgroup or endpoint could be extracted; otherwise, they were retained solely as supportive qualitative evidence and excluded from CIN2+-specific estimates. The review question, eligibility criteria, and extraction fields were defined before extraction, but the protocol was not prospectively registered in PROSPERO, OSF, or another registry. This absence is acknowledged as a limitation. A completed PRISMA 2020 checklist is provided in Supplementary Table S4.

### 2.2. Information Sources and Search Strategy

**Figure 1:**
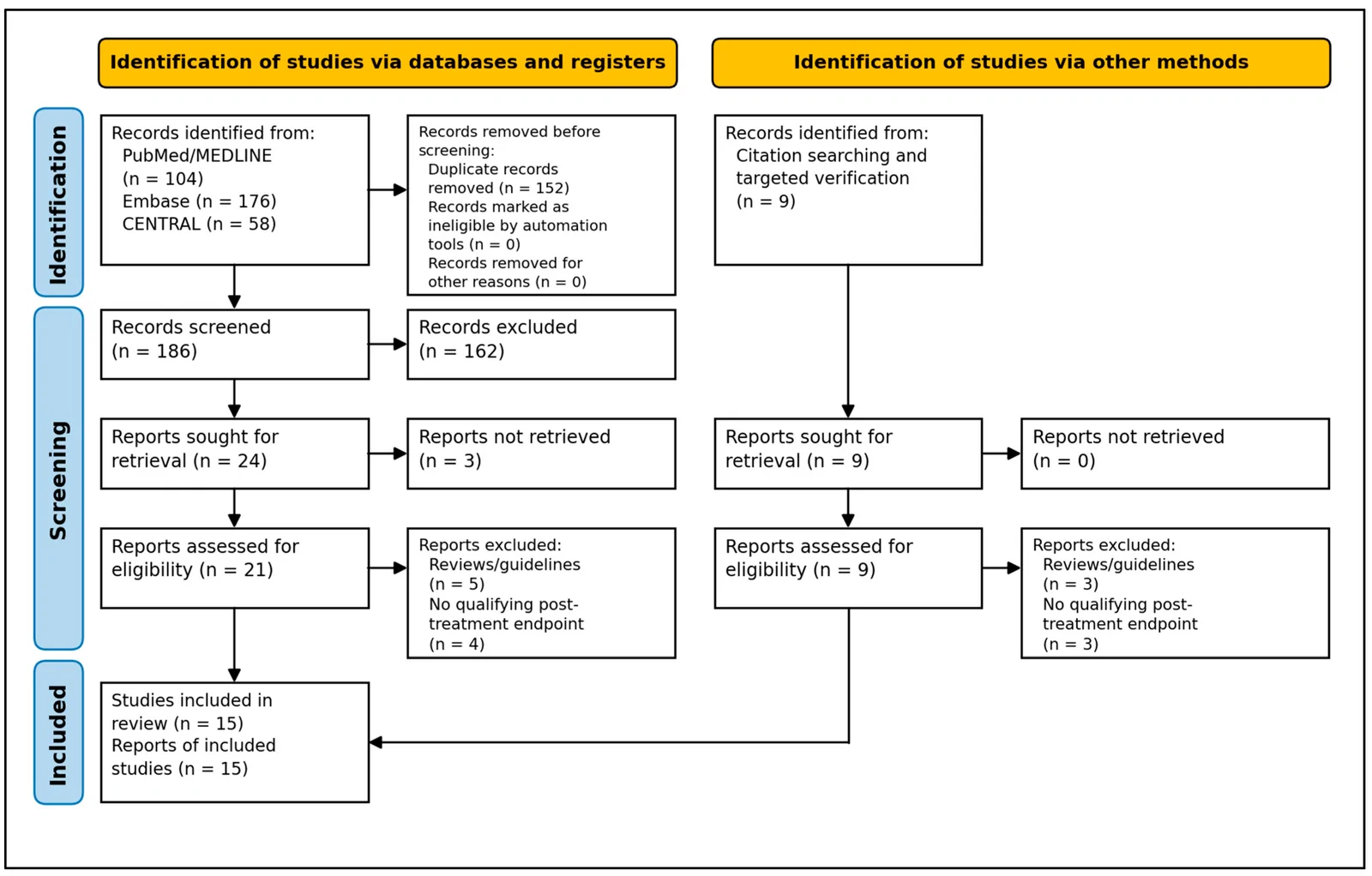
PRISMA 2020 flow diagram. The three unavailable reports are classified as reports not retrieved rather than as scientific exclusions. Targeted verification added the Heymans, Du, and Palmer studies to the qualitative synthesis. Supplementary Embase and CENTRAL searches conducted during peer review (28 August 2026) retrieved 234 records, of which 82 were unique; none met the eligibility criteria, and the counts shown incorporate these records.

The formal database search used PubMed/MEDLINE. The final verification search was completed on 24 August 2026 without language or publication-date limits. The complete reproducible query, including MeSH terms, free-text terms, field tags, parentheses, and Boolean structure, is provided in Supplementary Table S1. Query refinement was based on the breadth and relevance of retrieved titles, not on study results or anticipated findings. Reference lists of eligible studies and the Kocken, Arbyn, and Bomans reviews were checked systematically, and targeted verification recovered three additional relevant primary studies (Heymans, Du, and Palmer) [10,18,19]. In response to peer review, supplementary searches of Embase and the Cochrane Central Register of Controlled Trials (CENTRAL) were conducted on 28 August 2026, using strategies adapted from the PubMed/MEDLINE query and applying the same eligibility criteria (Supplementary Table S1). These searches retrieved 176 and 58 records, respectively; after removal of 152 duplicate records already identified in PubMed/MEDLINE, 82 new records were screened, four additional full texts were assessed, and no further eligible primary studies were identified. The PRISMA flow diagram (Figure 1) and Supplementary Table S1 were updated accordingly. Scopus and Web of Science were not searched, and this remains a limitation.

### 2.3. Eligibility Criteria and Study Selection

Studies were eligible if they: (1) enrolled women treated for histologically confirmed CIN2, CIN3, or HSIL, or reported a mixed-grade cohort from which a high-grade subgroup could be extracted; (2) performed post-treatment hrHPV testing, alone or with cytology; and (3) reported a histological or validated registry outcome. Mixed-grade or virological cohorts without an extractable high-grade denominator were included only for supportive qualitative synthesis and did not contribute to CIN2+-specific rates. Reviews, guidelines, primary-screening studies, and reports lacking a relevant post-treatment endpoint were excluded. Full-text accessibility was not treated as a scientific eligibility criterion. Three reports could not be retrieved and were classified as “reports not retrieved” in the PRISMA flow diagram; the archived screening log did not retain sufficient bibliographic detail to reconstruct their full references, and this reporting deficiency is disclosed in Supplementary Table S3. Title/abstract screening and full-text eligibility assessment were performed independently by G.T. and L.M.; disagreements were resolved by consensus. Agreement statistics were not prospectively planned or recorded.

### 2.4. Data Extraction and Items

G.T. extracted data into a predefined spreadsheet, and L.M. independently checked every included record and table entry against the source. Disagreements were resolved by consensus. Extracted fields included study design, treated and analysed cohorts, treatment, lesion grade, follow-up, outcome definition and grade, high-grade and any-grade event counts, risk factors, and diagnostic-accuracy measures. Treated and analysed denominators were recorded separately. Values were transcribed as reported; simple proportions and Wilson 95% confidence intervals were calculated by the review authors only when explicitly labelled. Studies without a recoverable CIN2+/CIN3+/HSIL+ denominator were marked “NR” and excluded from high-grade descriptive ranges.

### 2.5. Risk-of-Bias and Certainty Assessment

Risk of bias was assessed independently by G.T. and L.M. using QUADAS-2 [20] for the diagnostic-accuracy contribution of each study. The four domains were patient selection, index test, reference standard, and flow and timing; signalling questions and judgement rules are provided in Supplementary Table S2. Disagreements were resolved by consensus. QUADAS-2 judgements do not constitute a formal assessment of prognostic-factor analyses such as margin status, viral load, genotype, or platelet-to-lymphocyte ratio; those findings are therefore interpreted as exploratory. Formal GRADE certainty ratings were not applied because the review combined diagnostic-accuracy, prognostic, and registry questions in a narrative framework.

### 2.6. Synthesis and Statistical Presentation

A narrative synthesis was retained because only a small subset of studies provided complete and sufficiently comparable 2 × 2 data for the same index-test threshold, follow-up interval, outcome grade, and reference standard. Heterogeneity alone was not considered a reason to avoid meta-analysis. A limited forest plot presents study-specific high-grade event proportions with Wilson 95% confidence intervals, without a pooled diamond. Predictive values were interpreted alongside recurrence prevalence and verification procedures. The disease-free proportion after successful test of cure in Tan et al. was calculated by the review authors and is labelled as such; otherwise, accuracy estimates were transcribed from the source reports. Study-level reported diagnostic-accuracy estimates, together with an explicit statement of which two-by-two (TP, FP, FN, and TN) counts could not be retrieved, are summarised in Supplementary Table S5. An unpooled forest plot of sensitivity and specificity estimates is not presented because fewer than five studies reported these measures for a comparable test point, outcome grade, and verification approach.

## 3. Results

A total of fifteen primary studies were included in the qualitative synthesis (Figure 1) [10,18,19,21,22,23,24,25,26,27,28,29,30,31,32]. They enrolled 21,932 treated women; 18,992 contributed to the endpoint-specific analysed cohorts shown in Table 1. Denominators varied by outcome and follow-up stage and should not be interpreted as one uniform analytic population.

Table 1 summarises the fifteen studies. Four were conducted in Spain; the remaining European studies were from Belgium, Sweden, the United Kingdom, and Denmark. Four studies were from East Asia (three from China and one from South Korea), with one each from the United States and Australia. Five cohorts were prospective [10,21,22,26,29], eight were retrospective single-institution or regional studies [18,19,23,24,25,27,28,32], and two were registry-based [30,31]. The two registry cohorts contributed 13,652 of 21,932 treated women (62.2%), so statements based simply on the number of studies should not imply equal statistical weight. Denominator differences reflect loss to follow-up or endpoint-specific subsets: 45 women were lost in Lubrano, 260 of 330 underwent 6-month co-testing in Asciutto, 213 of 2729 formed the Kalampokas hrHPV-positive/cytology-negative subset, and 2253 of 8478 completed successful ToC in Tan. The reason for the 220-to-210 difference in Oncins Torres was not recoverable and is labelled NR rather than assumed.

**Figure 2:**
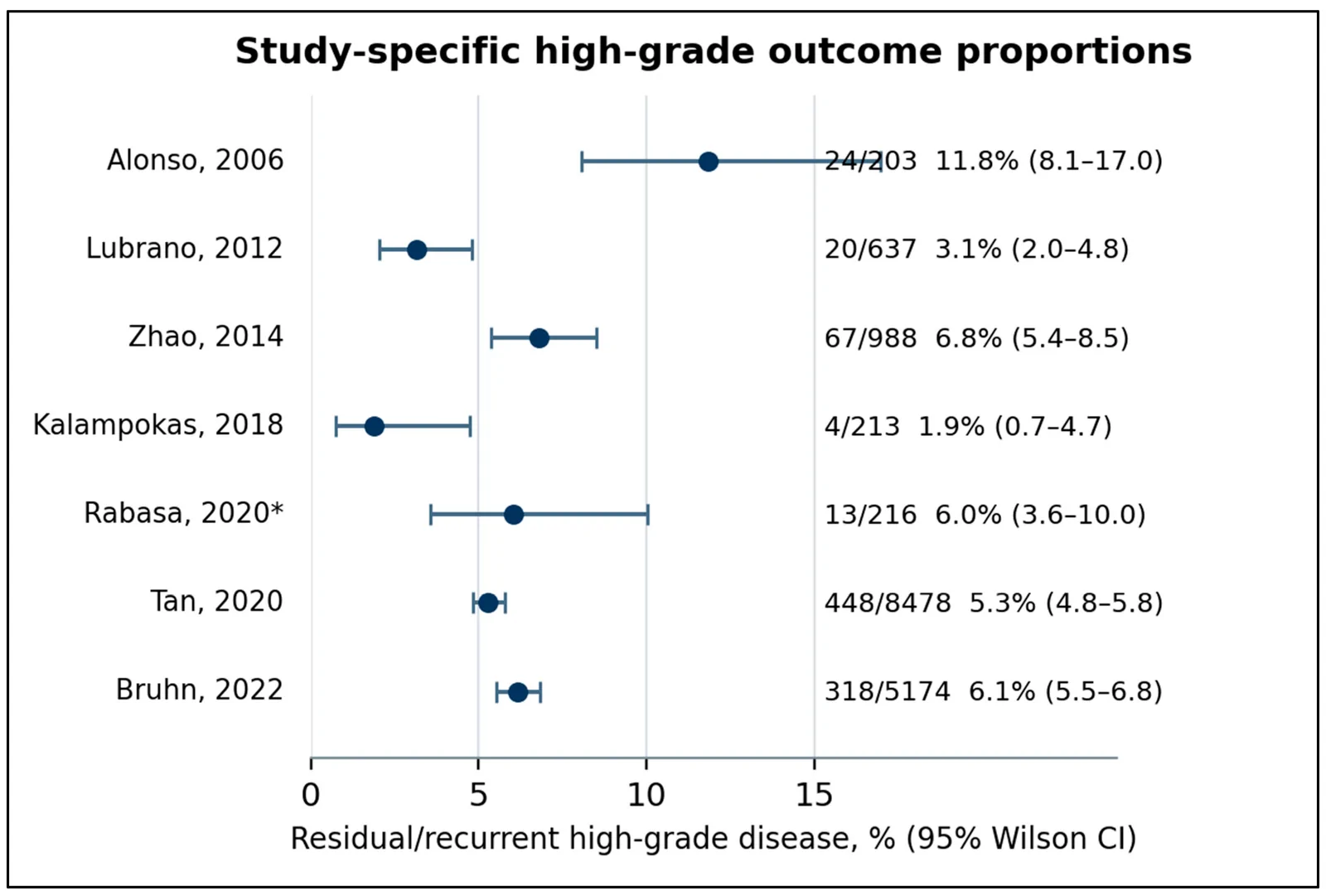
Study-specific proportions of residual/recurrent high-grade disease with 95% Wilson confidence intervals. No pooled estimate is presented because outcome ascertainment and follow-up were not sufficiently comparable. Proportions are shown for the studies reporting explicit high-grade denominators: Alonso [21], Lubrano [23], Zhao [24], Kalampokas [27], Rabasa [29], Tan [30], and Bruhn [31]. * For Rabasa, the event count (13/216) and its Wilson confidence interval were derived by the review authors from the reported 6.0% recurrence proportion and the analysed cohort of 216 women; the source did not report the absolute count.

Table 2 separates high-grade outcomes from any-grade CIN, hrHPV persistence, and composite endpoints. Explicit high-grade rates were 1.9–6.8% in most cohorts [24,27,29,30,31], while Alonso reported 11.8% (24/203) high-grade disease or carcinoma in an earlier intensively verified cohort [21]. By contrast, higher figures in Jeong, Lubrano, Mo, and Kalampokas represented any-grade or insufficiently grade-specific outcomes and were not labelled CIN2+ [22,23,25,27]. Post-treatment hrHPV positivity and involved margins were the most recurrent predictors, but margin location and processing varied. Type-specific persistence improved specificity in Heymans [10], and HPV-16 was associated with recurrence in Rabasa [29]; Oncins Torres did not find a statistically significant difference between HPV-16/18 and other high-risk types [28]. Genotype-specific persistence is therefore a promising, not definitively superior, risk stratifier. Figure 2 displays the study-specific high-grade proportions without pooling.

Table 3 shows that directly comparable diagnostic-accuracy measures were available for only a subset of studies. Within that subset, hrHPV testing was generally more sensitive than cytology, but the large number of NR cells precludes claiming uniform superiority across all included studies. Alonso reported hrHPV sensitivity of 97.2% versus 83.3% for a single cytology [21]; Heymans reported 100% sensitivity for pooled high/intermediate-risk HPV and 60% sensitivity with greater specificity for type-specific persistence [10]; and Bruhn reported 83.7% sensitivity and 98.6% NPV for hrHPV alone [31]. The Jeong source reports 100% sensitivity for hrHPV alone but 82% for combined testing; because the combination rule and denominator could not be reconciled, the value is retained with a cautionary footnote rather than interpreted as conventional either-positive co-testing [22]. Very high NPVs partly reflect the low 1.9–6.8% high-grade recurrence prevalence and may be inflated when test-negative women were not biopsied. Sensitivity and absolute post-test risk, such as the 0.8% risk after a negative test reported by Arbyn et al. [15], are more transportable than NPV alone. Study-level reported sensitivity, specificity, PPV, and NPV values, with confidence intervals where available, are tabulated in Supplementary Table S5, which also states explicitly for which studies the underlying 2 × 2 counts could not be retrieved.

Five studies—Alonso [21], Heymans [10], Asciutto [26], the protocol-driven prospective cohort of Rabasa [29], and the Danish registry cohort of Bruhn [31]—were judged at an overall low risk of bias for their diagnostic-accuracy contribution. Eight were rated unclear and two high. The Australian registry cohort of Tan [30] was rated unclear because follow-up testing was opportunistic rather than protocol-driven. Flow and timing were the most frequent concerns: selective colposcopy or biopsy in test-positive women can inflate sensitivity and NPV. Palmer [19] and Kalampokas [27] were rated high overall because verification was concentrated in test-positive or selected subsets. In Lubrano [23], Du [18], Zhao [24], and Tan [30], the high rating was confined to the flow-and-timing domain; in accordance with the pre-specified rule described in Supplementary Table S2 and in the footnote to Table 4, these studies were therefore assigned an unclear rather than high overall judgement. These QUADAS-2 judgements do not validate the separate prognostic associations reported for margins, genotype, viral load, or inflammatory markers.

## 4. Discussion

### 4.1. Analysis of Findings

The principal finding is that post-treatment hrHPV testing supports risk stratification after excisional treatment, but outcome grade and verification procedures materially affect the apparent performance. In studies reporting comparable CIN2+/CIN3+/HSIL+ outcomes, most event rates were approximately 2–7%, with one earlier cohort reporting 11.8%. Negative-test risks were low, but NPVs approaching 100% must be interpreted in the context of low recurrence prevalence and selective histological verification. Similar reassurance after negative post-treatment hrHPV testing has been reported in other observational follow-up series [33,34]. A negative hrHPV result may justify reduced-intensity post-treatment surveillance according to applicable guidelines [35]; it does not by itself justify discharge to routine population screening because treated women retain long-term excess cancer risk.

The present review should be interpreted alongside three major quantitative syntheses. Kocken et al. reported pooled sensitivities of 0.92 for hrHPV testing, 0.79 for cytology, and 0.95 for co-testing [14]. Arbyn et al. included 97 studies and 44,446 women and found 91.0% sensitivity and a 0.8% CIN2+ risk after a negative test [15]. Bomans et al. updated the evidence through August 2024, including 46 studies and 20,385 women; pooled hrHPV sensitivity and specificity were 86.8% and 80.5%, and treatment failure occurred in 6.6% [16]. The added value of the present review is not a larger pooled estimate, but transparent study-level separation of high-grade from any-grade outcomes, explicit denominator reconciliation, and focused discussion of margin-stratified registry data and newer biomarker cohorts.

Resection-margin status remains clinically relevant, but “positive margins” are not uniform: endocervical versus ectocervical involvement, excisional technique, specimen fragmentation, cautery artefact, and pathology processing can alter classification and risk. Margin-stratified hrHPV strategies are promising, but the suggestion that cytology can be omitted in women with free margins and negative hrHPV relies heavily on limited observational evidence and requires prospective validation. Type-specific persistence may raise specificity and PPV [10], and HPV-16 was associated with recurrence in Rabasa [29]; however, Oncins Torres did not show a significant excess risk for HPV-16/18 versus other high-risk types [28]. Genotyping should therefore be framed as an additional candidate risk stratifier rather than a firmly established superior predictor.

Adjunctive markers such as p16/Ki-67, methylation, HPV mRNA, and the platelet-to-lymphocyte ratio may improve triage of hrHPV-positive women, but evidence from the included studies is sparse, and these approaches require validation [32,36]. Smoking has been associated with longer cervical oncogenic HPV persistence and a lower probability of clearance in a prospective cohort of women [37]. The long-term excess risk of invasive cancer after treatment supports prolonged surveillance [38,39], whereas the obstetric consequences of repeated excision support avoiding unnecessary re-treatment [12]. Self-sampling and peri-treatment vaccination are relevant future directions [40,41,42], but they are ancillary to the present review question.

### 4.2. Study Limitations

This review has important limitations. The formal search was initially limited to PubMed/MEDLINE; structured citation checking recovered three missed primary studies, and supplementary Embase and CENTRAL searches conducted during peer review identified no additional eligible studies, but Scopus and Web of Science were not searched, so a residual risk of incomplete retrieval remains. The protocol was not prospectively registered. Three reports were not retrieved, and their full bibliographic details were not preserved in the archived log, introducing possible access and selection bias. Outcome heterogeneity was substantial: some cohorts reported CIN2+/CIN3+/HSIL+, whereas others reported any-grade CIN, hrHPV persistence, or composites including CIN1. Treated and analysed denominators also varied. Only a small subset provided comparable 2 × 2 data, precluding a reliable bivariate or HSROC model; the forest plot is descriptive and unpooled. Selective verification of test-negative women may overestimate sensitivity and NPV, and NPV is prevalence-dependent. QUADAS-2 was applied only to diagnostic-accuracy contributions, not to prognostic-factor analyses, and no formal GRADE assessment was undertaken. Formal publication-bias tests were not informative with few heterogeneous studies, but small studies with inconclusive or unfavourable results may be underrepresented. Finally, the two large registry cohorts account for most participants, so a count of studies does not reflect their unequal quantitative weight.

## 5. Conclusions

This focused review supports post-treatment hrHPV testing as a sensitive component of risk-based surveillance after excisional treatment for CIN2–3/HSIL. When reported separately, most explicit high-grade event rates were approximately 2–7%, while higher figures often reflected any-grade or composite outcomes. A negative hrHPV result is reassuring, but the magnitude of reassurance is influenced by low recurrence prevalence and incomplete histological verification. Negative hrHPV testing, particularly with free margins, may support reduced-intensity post-treatment surveillance under established guidelines; it should not be equated with unqualified return to routine population screening. Cytology omission and genotype- or margin-stratified pathways remain promising strategies that require prospective validation with harmonised CIN2+ endpoints, standardised assays, complete verification, and transparent reporting.

## Figures and Tables

**Table 1:** Characteristics and denominators of the fifteen included studies. Treated and analysed cohorts are shown separately. LEEP, loop electrosurgical excision procedure; LLETZ, large loop excision of the transformation zone; CIN, cervical intraepithelial neoplasia; HSIL, high-grade squamous intraepithelial lesion; ToC, test of cure; NR, not reported.

#	Study (Year)	Country/Design	Treatment	Treated Cohort	Analysed Cohort	Lesion Grade	Follow-Up	Ref
1	Alonso, 2006	Spain/prospective	LEEP	203	203	CIN2–3	6–12 mo	[21]
2	Jeong, 2009	South Korea/prospective	Conization + LEEP	95	95	High-grade CIN	3–24 mo	[22]
3	Heymans, 2011	Belgium/prospective	Conization	90	90	CIN; HG outcome	Post-treatment	[10]
4	Lubrano, 2012	Spain/retrospective	LLETZ	682	637	CIN2–3	Mean 39.9 mo	[23]
5	Du, 2013	China/retrospective	LEEP	141	141	HR-HPV-associated CIN	3–12 mo	[18]
6	Zhao, 2014	USA/retrospective	Excision	988	988	CIN2–3	Mean 36 mo	[24]
7	Mo, 2015	China/retrospective	LEEP	158	158	CIN2–3	3–24 mo	[25]
8	Palmer, 2016	UK/retrospective	LLETZ	2093	1794 at ToC	Any-grade CIN	6 mo	[19]
9	Asciutto, 2016	Sweden/prospective	LEEP	330	260 at 6 mo	Dysplasia incl. HG	6–36 mo	[26]
10	Kalampokas, 2018	UK/retrospective	Excision	2729	213 subset	Any-grade CIN; HG outcome	Mean 30.5 mo	[27]
11	Oncins Torres, 2018	Spain/retrospective	LLETZ	220	210; reason NR	HSIL/CIN2+	To 2017	[28]
12	Rabasa, 2020	Spain/prospective	LEEP	216	216	HSIL (CIN2/3)	Intraop, 6, 12 mo	[29]
13	Tan, 2020	Australia/registry	Excision	8478	8478; 2253 ToC	HSIL	Up to 5 y	[30]
14	Bruhn, 2022	Denmark/registry	Conization	5174	5174	CIN2+	3 y	[31]
15	Huang, 2023	China/retrospective	LEEP	335	335	HSIL (CIN2/3)	3–5 y	[32]

**Table 2:** Outcome grade, high-grade event rates, supportive outcomes, and principal predictors. High-grade rates are reported only when a CIN2+/CIN3+/HSIL+ denominator was recoverable. OR, odds ratio; HR, hazard ratio; CI, confidence interval; RLU, relative light units; PLR, platelet-to-lymphocyte ratio; NR, not reported.

#	Study (Year)	Outcome Grade	High-Grade Rate	Any-Grade/Supportive Outcome	Principal Predictors	Ref
1	Alonso, 2006	HSIL/CIN2+ or cancer	11.8% (24/203)	Any CIN: 17.7% (36/203)	High pre-LEEP hrHPV load; positive margins	[21]
2	Jeong, 2009	Any CIN on biopsy	NR	Any CIN: 17.9% (17/95)	Positive margins; post-treatment hrHPV load > 100 RLU	[22]
3	Heymans, 2011	Residual HSIL/CIN2+	NR	Type-specific persistence increased PPV	Persistent same hrHPV genotype	[10]
4	Lubrano, 2012	High-grade CIN	3.1% (20/637)	Any CIN: 13.9% (88/637)	Post-treatment hrHPV; positive margins	[23]
5	Du, 2013	HPV persistence/recurrent CIN	NR	HPV persistence: 44.6%, 10.6%, 5.7%, 2.1% at 3, 6, 9, 12 mo	Persistent hrHPV; HPV-16 cleared more slowly	[18]
6	Zhao, 2014	CIN2/3	6.8% (67/988)	67.2% within 2 y	CIN3; positive margins; hrHPV-positive follow-up	[24]
7	Mo, 2015	Grade not separated	NR	Residual/recurrent disease: 15.8% (25/158)	Persistent hrHPV; high viral load; positive margins	[25]
8	Palmer, 2016	Residual CIN; grade NR	NR	36 residual CIN after failed ToC; 298/1794 hrHPV+	Margin status did not correlate with hrHPV at ToC	[19]
9	Asciutto, 2016	HSIL	NR overall	No HSIL/cancer after negative 6-mo co-test; 40/260 hrHPV+	Positive hrHPV associated with cytological abnormality	[26]
10	Kalampokas, 2018	CIN2/3	1.9% (4/213)	Any CIN: 11.3% (24/213)	Selected hrHPV+/cytology-negative subgroup	[27]
11	Oncins Torres, 2018	hrHPV persistence	NR	Persistence: 6.7% (14/210)	No significant HPV-16/18 vs. other hrHPV difference	[28]
12	Rabasa, 2020	HSIL recurrence	6.0%	—	Intraoperative/6-mo hrHPV; HPV-16; margins	[29]
13	Tan, 2020	Histological HSIL+	5.3% (448/8478)	1 recurrence after successful ToC (1/2253)	Failure to complete ToC	[30]
14	Bruhn, 2022	Histological CIN2+	6.1% (318/5174)	Free margins 2.6% vs. involved 10.2%	Involved margins; hrHPV+; ASC-US+ cytology	[31]
15	Huang, 2023	Composite included CIN1	NR	50 events including CIN1	hrHPV; high PLR; gland invasion	[32]

**Table 3:** Reported diagnostic performance of post-treatment hrHPV testing and comparators for high-grade disease. Sens, sensitivity; Spec, specificity; PPV, positive predictive value; NPV, negative predictive value; NR, not reported. Superscript notes explain the Jeong co-testing inconsistency and the author-calculated Tan value.

#	Study (Year)	Test Point	hrHPV Sens/Spec	hrHPV PPV/NPV	Cytology Sens	Co-Test/Stratified Strategy	Ref
1	Alonso, 2006	6–12 mo	97.2%/81.4%	NR/NR	83.3% single	hrHPV + cytology: Sens 100%, NPV 100%	[21]
2	Jeong, 2009 ^a^	6 mo	100%/NR	NR/100%	Significant at first visit	Reported combined Sens 82%, Spec 76%, NPV 95%	[22]
3	Heymans, 2011	Post-treatment	100%/86% pooled 13 hrHPV types	NR/100%	NR	Same-type persistence: Sens 60%, Spec 95%, PPV 43%	[10]
4	Lubrano, 2012	Post-treatment	NR/NR	NR/NR	NR	hrHPV + margins independently predictive	[23]
5	Du, 2013	3–12 mo	NR/NR	NR/NR	NR	Persistent hrHPV associated with recurrence	[18]
6	Zhao, 2014	Follow-up	NR/NR	NR/NR	NR	CIN2/3: 14.5% hrHPV+ vs. 1.7% hrHPV−	[24]
7	Mo, 2015	6–18 mo	NR/NR	NR/100% double-negative	NR	No recurrence when persistently double-negative	[25]
8	Palmer, 2016	6 mo	NR/NR	NR/NR	NR	Selective colposcopy after failed ToC	[19]
9	Asciutto, 2016	6 mo	NR/NR	NR/100% HSIL	NR	Negative co-test NPV 100% (95% CI 99.8–100)	[26]
10	Kalampokas, 2018	6 mo	NR/NR	NR/NR	NR	hrHPV+/cytology−: 1.9% CIN2/3	[27]
11	Oncins Torres, 2018	Post-treatment	NR/NR	NR/NR	NR	hrHPV persistence 6.7%	[28]
12	Rabasa, 2020	Intraoperative	85.7%/80.8%	24.0%/98.8%	NR	6-mo hrHPV: Sens 76.9%, NPV 98.0%	[29]
13	Tan, 2020 ^b^	ToC	NR/NR	NR/99.96% disease-free ^b^	NR	Successful ToC: 1 recurrence/2253	[30]
14	Bruhn, 2022	~6 mo	83.7%/75.4%	18.3%/98.6%	66.8%	Margin + hrHPV: Sens 95.9%, NPV 99.4%	[31]
15	Huang, 2023	3–5 y	NR/NR	NR/NR	NR	hrHPV HR 3.901; PLR HR 2.082	[32]

^a^ Jeong et al. reported lower sensitivity for the combined result than for hrHPV alone; the source does not provide sufficient information to reconcile the combination rule or denominator. ^b^ Tan disease-free proportion calculated by the review authors as 1 − (1/2253); it was not reported as a conventional diagnostic NPV.

**Table 4:** QUADAS-2 risk-of-bias assessment for the diagnostic-accuracy contribution of each included study. The Table does not formally assess prognostic-factor analyses. RoB, risk of bias; NA, not applicable.

#	Study (Year)	Patient Selection	Index Test	Reference Standard	Flow & Timing	Overall RoB	Ref
1	Alonso, 2006	Low	Low	Low	Unclear	Low	[21]
2	Jeong, 2009	Low	Unclear	Low	Unclear	Unclear	[22]
3	Heymans, 2011	Low	Low	Low	Unclear	Low	[10]
4	Lubrano, 2012	Unclear	Low	Low	High	Unclear	[23]
5	Du, 2013	Unclear	Low	Unclear	High	Unclear	[18]
6	Zhao, 2014	Unclear	Low	Unclear	High	Unclear	[24]
7	Mo, 2015	Unclear	Low	Low	Unclear	Unclear	[25]
8	Palmer, 2016	Unclear	Low	High	High	High	[19]
9	Asciutto, 2016	Low	Low	Unclear	Low	Low	[26]
10	Kalampokas, 2018	Unclear	Low	High	High	High	[27]
11	Oncins Torres, 2018	Unclear	Unclear	Unclear	Unclear	Unclear	[28]
12	Rabasa, 2020	Low	Low	Low	Low	Low	[29]
13	Tan, 2020	Low	Low	Low	High	Unclear	[30]
14	Bruhn, 2022	Low	Low	Low	Low	Low	[31]
15	Huang, 2023	Unclear	Low	Low	Unclear	Unclear	[32]

Derivation of the overall judgement: the overall risk of bias was rated High when at least one of the three critical domains (patient selection, index test, or reference standard) was rated High; a High rating confined to the flow-and-timing domain, with no critical domain rated High, resulted in an Unclear overall judgement, because incomplete or opportunistic verification limits confidence in, but does not necessarily invalidate, the accuracy estimates. This rule applies to Lubrano, Du, Zhao, and Tan, which remain Unclear overall despite a High flow-and-timing rating.

## Data Availability

No new data were created or analyzed in this study. Data sharing is not applicable to this article.

## Supplementary Materials

The following supporting information can be downloaded at: https://www.mdpi.com/article/10.3390/medsci14050561/s1; Table S1: Complete PubMed/MEDLINE search strategy and supplementary Embase and CENTRAL search strategies; Table S2: QUADAS-2 signalling questions and judgement rules; Table S3: Reports not retrieved; Table S4: PRISMA 2020 checklist; Table S5: Study-level reported diagnostic-accuracy estimates and availability of 2 × 2 counts.
