# Supplementary material for: Post-Treatment High-Risk HPV Testing as a “Test of Cure”: A Systematic Review
Source: Med Sci (Basel). 2026 Sep 11;14(5):561. doi: 10.3390/medsci14050561 (PMC13609480; doi:10.3390/medsci14050561)
Supplement: Supplementary file 1 [file medsci-14-00561-s001.zip › medsci-4481408-supplementary.pdf]

Supplementary Materials

The following supplementary material is embedded in this revised manuscript: full PubMed/MEDLINE strategy with the supplementary Embase and CENTRAL strategies, risk-of-bias judgement rules, unavailable-report disclosure, and the completed PRISMA 2020 checklist, together with study-level reported diagnostic-accuracy estimates (Table S5).

Table S1. Complete PubMed/MEDLINE search strategy and supplementary Embase and CENTRAL search strategies

Source / date	Verbatim strategy or method
PubMed/MEDLINE; final verification 24 August 2026	(("Papillomavirus Infections"[MeSH Terms] OR "human papillomavirus"[Title/Abstract] OR HPV[Title/Abstract] OR hrHPV[Title/Abstract]) AND ("Cervical Intraepithelial Neoplasia"[MeSH Terms] OR CIN2[Title/Abstract] OR CIN3[Title/Abstract] OR HSIL[Title/Abstract]) AND ("Conization"[MeSH Terms] OR conization[Title/Abstract] OR conisation[Title/Abstract] OR LEEP[Title/Abstract] OR LLETZ[Title/Abstract] OR excision[Title/Abstract]) AND ("test of cure"[Title/Abstract] OR follow-up[Title/Abstract] OR post-treatment[Title/Abstract] OR residual[Title/Abstract] OR recurrent[Title/Abstract] OR recurrence[Title/Abstract] OR persistence[Title/Abstract] OR "treatment failure"[Title/Abstract]))
Embase (Elsevier); supplementary search 28 August 2026	Strategy adapted from the PubMed/MEDLINE query using Emtree and free-text terms: ('papillomavirus infection'/exp OR 'human papillomavirus':ti,ab OR hpv:ti,ab OR hrhpv:ti,ab) AND ('uterine cervix dysplasia'/exp OR cin2:ti,ab OR cin3:ti,ab OR hsil:ti,ab) AND ('conization'/exp OR conization:ti,ab OR conisation:ti,ab OR leep:ti,ab OR lletz:ti,ab OR excision:ti,ab) AND ('test of cure':ti,ab OR 'follow up':ti,ab OR 'post treatment':ti,ab OR residual:ti,ab OR recurrent:ti,ab OR recurrence:ti,ab OR persistence:ti,ab OR 'treatment failure':ti,ab). No language or date limits. 176 records retrieved; no additional eligible studies identified.
CENTRAL (Cochrane Library); supplementary search 28 August 2026	Same four concept blocks as the PubMed/MEDLINE query, applied to title/abstract/keyword fields. No language or date limits. 58 records retrieved; no additional eligible studies identified.
Citation checking	Backward and forward citation checking of eligible primary studies and the Kocken 2012, Arbyn 2017, and Bomans 2025 reviews; reviewer-specified Palmer, Heymans, and Du reports were verified.
Limits	No language or publication-date limits at identification. No outcome-based narrowing. Scopus and Web of Science were not formally searched; Embase and CENTRAL were searched during peer review as described above.

Table S2. QUADAS-2 signalling questions and judgement rules

Domain	Key questions	Low / unclear / high judgement
Patient selection	Consecutive/random sampling; inappropriate exclusions; case-control design	Low if representative with appropriate exclusions; unclear if reporting incomplete; high if selected subset or inappropriate exclusions
Index test	Pre-specified threshold; interpretation without reference-standard knowledge	Low if threshold and interpretation were predefined; unclear if blinding/threshold reporting incomplete; high if data-driven

Domain	Key questions	Low / unclear / high judgement
Reference standard	Capacity to identify CIN2+; interpretation without index-test knowledge	Low for uniform histology/validated registry; unclear if verification or blinding incomplete; high for selective/non-equivalent standards
Flow and timing	Appropriate interval; same reference standard; complete inclusion in analysis	Low if protocol driven and complete; unclear if losses/timing incompletely described; high with differential or selective verification
Overall	Domain synthesis	High if any critical domain (patient selection, index test, or reference standard) high; unclear if one or more domains unclear without critical high risk, including when a high rating is confined to flow and timing; low when all critical domains low and remaining concerns minor

Table S3. Reports not retrieved

Three reports sought during the original search could not be retrieved. Their complete bibliographic identifiers were not preserved in the archived screening log; consequently, the authors cannot provide verifiable full references and have not fabricated them. They are now classified as reports not retrieved in Figure 1. This documentation failure is acknowledged as a limitation.

Table S4. PRISMA 2020 checklist

Item	Topic	Where reported
1	Title	Title identifies the report as a systematic review.
2	Abstract	Structured summary includes sources, participants, results, risk of bias, and registration status.
3	Rationale	Introduction.
4	Objectives	End of Introduction.
5	Eligibility criteria	Section 2.3.
6	Information sources	Section 2.2 and Table S1.
7	Search strategy	Table S1; PubMed strategy reproduced verbatim.
8	Selection process	Section 2.3; two independent reviewers, consensus resolution.
9	Data collection process	Section 2.4; extraction and independent checking.
10	Data items	Section 2.4.
11	Risk-of-bias assessment	Section 2.5 and Table S2.
12	Effect measures	Section 2.6; proportions and reported diagnostic measures.
13	Synthesis methods	Section 2.6; narrative synthesis and unpooled forest plot.
14	Reporting-bias assessment	Section 4.2; formal testing not informative.
15	Certainty assessment	Section 2.5; formal GRADE not applied, with rationale.
16	Study selection	Figure 1; unavailable-report identification limitation disclosed.
17	Study characteristics	Table 1.

Item	Topic	Where reported
18	Risk of bias in studies	Table 4 and Results narrative.
19	Results of individual studies	Tables 2–3 and Figure 2.
20	Results of syntheses	Section 3; no pooled estimate.
21	Reporting biases	Section 4.2.
22	Certainty of evidence	Section 2.5 and Section 4.2.
23	Discussion	Section 4.
24	Registration and protocol	Section 2.1; not prospectively registered.
25	Support	Funding statement.
26	Competing interests	Conflicts of Interest statement.
27	Availability of data/materials	Data Availability Statement and embedded supplementary tables.

Table S5. Study-level reported diagnostic-accuracy estimates and availability of 2 × 2 counts

Table S5 summarises, for each included study, the diagnostic-accuracy estimates reported for the detection of residual/recurrent high-grade disease, transcribed verbatim from the source reports. Complete two-by-two counts (TP, FP, FN, TN) could not be retrieved from any published report, and confidence intervals are shown only where the source reported them; cells marked NR indicate values not reported in the source. Because fewer than five studies reported sensitivity and specificity for a comparable test point, outcome grade, and verification approach, an unpooled forest plot of accuracy estimates would not be informative and is not presented.

Study (year)	Test / time point	TP/FP/FN/TN	hrHPV Sens (95% CI)	hrHPV Spec (95% CI)	hrHPV PPV	hrHPV NPV (95% CI)	Cytology / co-test / other reported values
Alonso, 2006 [21]	6–12 mo	NR [c]	97.2% (CI NR)	81.4% (CI NR)	NR	NR	Cytology Sens 83.3%; hrHPV + cytology: Sens 100%, NPV 100%
Jeong, 2009 [22]	6 mo	NR [c]	100% (CI NR)	NR	NR	100% (CI NR)	Combined testing: Sens 82%, Spec 76%, NPV 95% (see Table 3, note a)
Heymans, 2011 [10]	Post-treatment	NR [c]	100% (CI NR; pooled hrHPV)	86% (CI NR)	NR	100% (CI NR)	Type-specific persistence: Sens 60%, Spec 95%, PPV 43%
Lubrano, 2012 [23]	Post-treatment	NR [c]	NR	NR	NR	NR	Accuracy measures not reported; hrHPV and margins independently predictive
Du, 2013 [18]	3–12 mo	NR [c]	NR	NR	NR	NR	Accuracy measures not reported
Zhao, 2014 [24]	Follow-up	NR [c]	NR	NR	NR	NR	Risk stratification only: CIN2/3 in 14.5% of hrHPV-positive vs 1.7% of hrHPV-negative women
Mo, 2015 [25]	6–18 mo	NR [c]	NR	NR	NR	100% (CI NR; persistently double-negative co-test)	No recurrence with persistently double-negative co-testing
Palmer, 2016 [19]	6 mo	NR [c]	NR	NR	NR	NR	Accuracy measures not reported
Asciutto, 2016 [26]	6 mo	NR [c]	NR	NR	NR	100% (95% CI 99.8–100; negative co-test)	—
Kalampokas, 2018 [27]	6 mo	NR [c]	NR	NR	NR	NR	Selected hrHPV-positive/cytology-negative subgroup; accuracy not estimable
Oncins Torres, 2018 [28]	Post-treatment	NR [c]	NR	NR	NR	NR	Accuracy measures not reported

Rabasa, 2020 [29]	Intraoperative	NR [c]	85.7% (CI NR)	80.8% (CI NR)	24.0%	98.8% (CI NR)	6-mo hrHPV: Sens 76.9%, NPV 98.0%
Tan, 2020 [30]	ToC	NR [c]	NR	NR	NR	99.96% disease-free (author-calculated; not a conventional NPV)	—
Bruhn, 2022 [31]	~6 mo	NR [c]	83.7% (CI NR)	75.4% (CI NR)	18.3%	98.6% (CI NR)	Cytology Sens 66.8%; margins + hrHPV: Sens 95.9%, NPV 99.4%
Huang, 2023 [32]	3–5 y	NR [c]	NR	NR	NR	NR	Hazard ratios only: hrHPV HR 3.901; PLR HR 2.082

Sens, sensitivity; Spec, specificity; PPV/NPV, positive/negative predictive value; ToC, test of cure; HR, hazard ratio; PLR, platelet-to-lymphocyte ratio; CI, confidence interval; NR, not reported. [c] The complete TP/FP/FN/TN cross-classification could not be retrieved from the published report; this is stated explicitly in accordance with the review protocol.
